# Implications for health and disease in the genetic signature of the Ashkenazi Jewish population

**DOI:** 10.1186/gb-2012-13-1-r2

**Published:** 2012-01-25

**Authors:** Saurav Guha, Jeffrey A Rosenfeld, Anil K Malhotra, Annette T Lee, Peter K Gregersen, John M Kane, Itsik Pe'er, Ariel Darvasi, Todd Lencz

**Affiliations:** 1 Department of Psychiatry, Division of Research, The Zucker Hillside Hospital Division of the North Shore - Long Island Jewish Health System, 75-59, 263rd St Glen Oaks, NY 11004, USA; 2 Center for Psychiatric Neuroscience, The Feinstein Institute for Medical Research, 350 Community Drive, Manhasset, NY 11030, USA; 3 Department of Psychiatry and Behavioral Science, Albert Einstein College of Medicine of Yeshiva University, 1300 Morris Park Ave, Bronx, NY 10461, USA; 4 Department of Psychiatry, Hofstra University School of Medicine, Hempstead, NY 11549, USA; 5 Department of Molecular Medicine, Hofstra University School of Medicine, Hempstead, NY 11549, USA; 6 Robert S Boas Center for Human Genetics and Genomics, The Feinstein Institute for Medical Research, 350 Community Drive, Manhasset, NY 11030, USA; 7 Department of Computer Science, Columbia University, 500 W 120th St New York, NY 10027, USA; 8 Department of Genetics The Institute of Life Sciences, The Hebrew University of Jerusalem, Givat Ram, Jerusalem, 91904, Israel

## Abstract

**Background:**

Relatively small, reproductively isolated populations with reduced genetic diversity may have advantages for genomewide association mapping in disease genetics. The Ashkenazi Jewish population represents a unique population for study based on its recent (< 1,000 year) history of a limited number of founders, population bottlenecks and tradition of marriage within the community. We genotyped more than 1,300 Ashkenazi Jewish healthy volunteers from the Hebrew University Genetic Resource with the Illumina HumanOmni1-Quad platform. Comparison of the genotyping data with that of neighboring European and Asian populations enabled the Ashkenazi Jewish-specific component of the variance to be characterized with respect to disease-relevant alleles and pathways.

**Results:**

Using clustering, principal components, and pairwise genetic distance as converging approaches, we identified an Ashkenazi Jewish-specific genetic signature that differentiated these subjects from both European and Middle Eastern samples. Most notably, gene ontology analysis of the Ashkenazi Jewish genetic signature revealed an enrichment of genes functioning in transepithelial chloride transport, such as *CFTR*, and in equilibrioception, potentially shedding light on cystic fibrosis, Usher syndrome and other diseases over-represented in the Ashkenazi Jewish population. Results also impact risk profiles for autoimmune and metabolic disorders in this population. Finally, residual intra-Ashkenazi population structure was minimal, primarily determined by class 1 MHC alleles, and not related to host country of origin.

**Conclusions:**

The Ashkenazi Jewish population is of potential utility in disease-mapping studies due to its relative homogeneity and distinct genomic signature. Results suggest that Ashkenazi-associated disease genes may be components of population-specific genomic differences in key functional pathways.

## Background

Since the advent of genomewide SNP microarrays for disease mapping, considerable attention has been paid to the potentially confounding role of population stratification [1,2]. In addition to variation introduced by major continental ancestry, substantial intra-continental clines have been reliably demonstrated, typically mapping onto geographic patterns of historic migration [3-5]. By contrast, population isolates and relatively small founder populations demonstrate less background diversity, which may provide increased power to detect disease-related alleles [6,7]. Nevertheless, even these populations tend to reveal very subtle patterns of genetic structure that reflect demographic history and may affect interpretation of disease association studies [8-10].

The Ashkenazi Jewish (AJ) population is one such founder cohort, composed of Jewish individuals whose ancestors are thought to have advanced from the Rhine valley to populate Eastern Europe and beyond, beginning approximately 1,000 years ago [11]. The AJ population has been associated with very specific genetically derived predispositions to disease, primarily monogenic recessive disorders [12], but more recent studies also demonstrate increased frequency of certain alleles associated with complex diseases [13-15]. Despite the interest in the AJ population for disease mapping, however, population genetic studies in AJ cohorts to date have not focused on the relevance of genetic results to the study of complex disease.

Classic population genetic studies of Jewish cohorts, based on uniparental markers, have provided strong evidence of founder effects for the AJ population in both the mitochondrial and Y-chromosome lineage [16,17]. Such studies typically have shown reduced variability within AJ samples, and a greater degree of resemblance to other Levantine-derived populations (including Arabs and non-Ashkenazi Jews) than to the host European populations; moreover, these studies have concluded that genetic drift has played a primary role in the heightened frequency of certain parental lineages that are rare or virtually absent in other populations [18,19]. More recent studies have also demonstrated the ability of SNP microarrays to differentiate AJ samples embedded within larger non-AJ European-American cohorts [20-22]; these studies placed AJ samples along a dimension intermediate to European and Middle Eastern populations. Most recently, three genomewide studies of autosomal markers in Jewish samples of varying origins have yielded results indicating: 1) considerable similarity between AJ and (most) non-Ashkenazi Jewish cohorts; and 2) Jewish populations (except those from India and Ethiopia) can be viewed as a mixture of European and Middle Eastern genetic ancestry [23-25]. However, two of these studies [23,24] were limited to relatively small sample sizes of AJ individuals, which may have restricted their ability to detect AJ-specific patterns of genetic variation. Moreover, these studies did not specifically test for geographic or other structure within the AJ population, and no attempt was made to characterize AJ-related variation with respect to disease susceptibility.

The present study was designed to examine these issues using genomewide SNP markers in a very large (*n *= 1,394) cohort of unselected AJ individuals from Israel. First, we sought to identify an AJ-specific allelic pattern from autosomal markers, using both clustering and principal components approaches as applied to AJ samples and non-Jewish samples derived from European, Middle Eastern, and Central/South Asian origins. Next, we tested whether genetic distance measures placed AJ in an intermediate position relative to European and Middle Eastern populations. Additionally, we examined whether the AJ population demonstrated internal structure, and whether any such structure would correlate with geographical region of origin. Next, we used genome wide association study (GWAS) methods to examine the relationship of AJ-specific variation to the biology of health and disease. Finally, we provide an optimized and cross-validated list of AJ-related ancestry informative markers (AIMs) for future disease-mapping studies.

## Results

### Ancestry estimation

**Figure 1:**
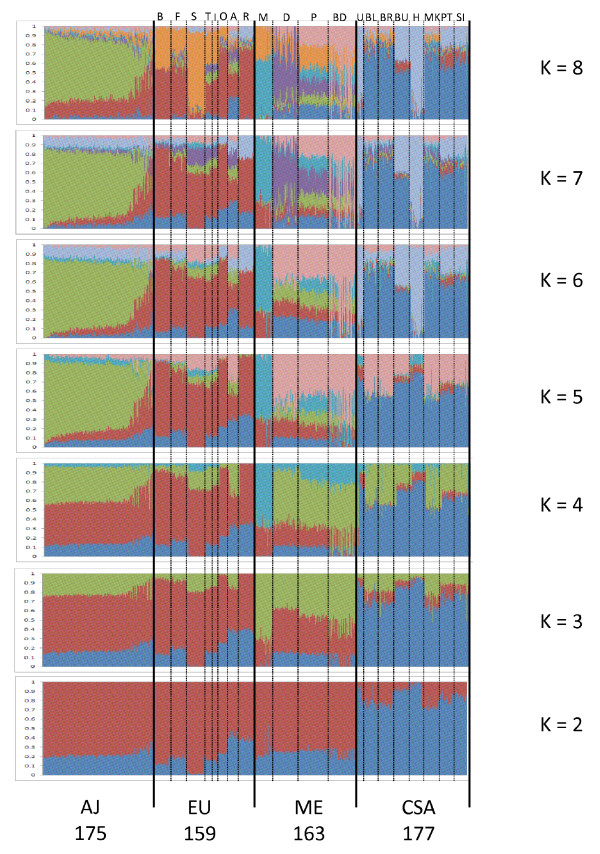
**ADMIXTURE analysis (K = 2 to 8) for ancestry estimation of the Ashkenazi Jewish (AJ) and three neighboring populations**. Three neighboring populations - Europeans (EU), Middle Easterners (ME), and Central/South Asians (CSA) - were derived from the HGDP. Each individual is represented by a thin vertical line, which is partitioned into K colored segments that represent the individual's estimated membership fractions in K clusters. Black lines separate major population groups based on geography. Geographical population groups and their respective N's are labeled below the figure. Specific ethnic subgroups are labeled above the figure: Europeans (B = Basque; F = French; S = Sardinian, T = Tuscan, I = Italian, O = Orcadian, A = Adygei, R = Russian); Middle Easterners (M = Mozabite, D = Druze, P = Palestinian, BD = Bedouin); and Central/South Asians (U = Uygur, BL = Balochi, BR = Brahui, BU = Burusho, H = Hazara, MK = Makrani, PT = Pathan, SI = Sindhi).

First, we utilized a clustering approach based on maximum likelihood estimation (ADMIXTURE software, details in Materials and methods) to detect underlying ancestral populations in AJ samples compared with members of three neighboring population groups derived from the human genome diversity panel (HGDP): European (EU; *n *= 159), Middle Eastern (ME; *n *= 163), and Central/South Asian (CSA; *n *= 177, excluding Kalash as per [23]). We initially selected *n *= 175 AJ subjects of varying national origins at random, in order to maintain roughly equal sample size with each of the other three groups. Approximately 95,600 unlinked SNPs were included in the analysis. Figure 1 demonstrates ADMIXTURE results for K = 2 to 8. By K = 5, AJ (pea green) are clearly differentiated from the other three major groups.

**Figure 2:**
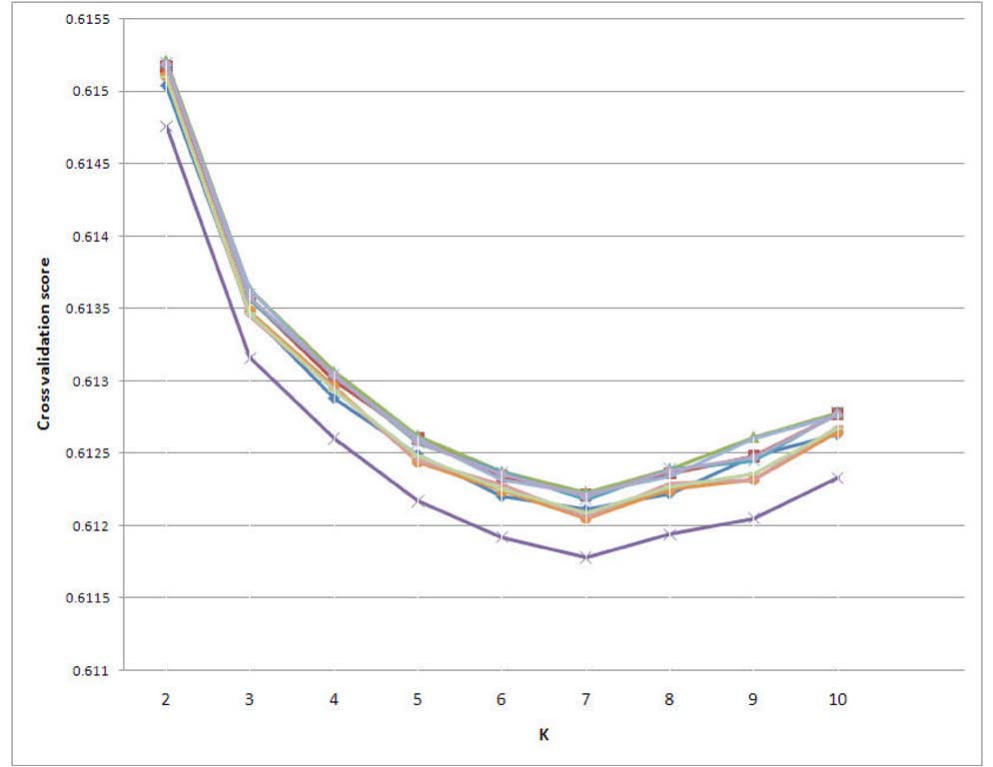
**Ten-fold cross-validation of K = 2 through K = 10 clusters from ADMIXTURE analysis with 10 randomly selected subsets of *n *= 175 individuals from the Ashkenazi Jewish (AJ) cohort, combined with HGDP subjects as indicated in Figure 1**. The x-axis represents the number of clusters (K) in the model and the y-axis represents cross-validation score (lower scores represent better fit of the model to the data). Ten different colors represent different runs.

Ten-fold cross-validation was performed with ten random AJ subsamples of *n *= 175, making use of our entire cohort, and the ADMIXTURE model fit was compared for K = 2 through K = 10. Figure 2 demonstrates that for each of the runs, the optimum cross-validation score was reached at K = 7; results for each of the ten AJ subsamples were nearly identical. Across the ten runs, the mean cross-validation score for the K = 7 solution (mean = 0.612113, standard deviation (SD) = 0.000135) was significantly smaller than the next lowest values (for K = 6 and K = 8: mean ± SD = 0.612265 ± 0.000135 and 0.612283 ± 0.000135, respectively; compared to K = 7 mean: t = 2.52, df = 18, *P *= 0.0215 and t = 2.82, df = 18, *P *= 0.0114, respectively).

As shown in Figure 1, for K = 7, pea green remains the dominant AJ color, accounting for as much as 87.5% of the ancestry of AJ individuals. Across all AJ samples, the median degree of contribution of this component was 64.6%; the mean (57.9 ± 19.1%) was somewhat lower than the median due to the presence of a number of subjects in the cohort with virtually no AJ contribution. Amongst non-AJ samples, sharing of this ancestry component was quite limited, with the greatest amount of overlap (approximately 10%) seen for Palestinians, Adygei (from the northern Caucasus), and Italians/Tuscans/Sardinians, respectively. At the same time, most of the AJ samples demonstrated little overlap with other specific ancestry components, with the exception of a subset of approximately 16% of the sample that had significant contributions from the European ancestry component (red). These admixed individuals will be examined in greater detail below.

Results did not change when we re-ran these ADMIXTURE analyses with larger subsamples of the Ashkenazi cohort (*n *= 350, *n *= 700, and *n *= 1,050 AJ individuals); for each of these analyses, K = 7 provided the optimal solution. Results changed slightly when the full Ashkenazi cohort was compared to the neighboring HGDP populations; as depicted in Additional file 1, the K = 8 solution was marginally (but not significantly) better than the K = 7 solution, which was also indistinguishable from the K = 9 solution. Compared to the K = 7 results, however, neither of these solutions introduced substantive changes into the AJ population ancestry component.

Additional file 3 further demonstrates similar results when all HGDP samples are included in the ADMIXTURE analysis. Cross-validation analysis (ten runs) indicated that model fit is optimized at K = 11, with second-best fit obtained at K = 8, which coincides with the emergence of the AJ-specific ancestry component (colored brown in Additional file 3). Moreover, at K = 11, there is virtually no evidence of this AJ component in any of the other populations.

### Principal components analysis

**Figure 3:**
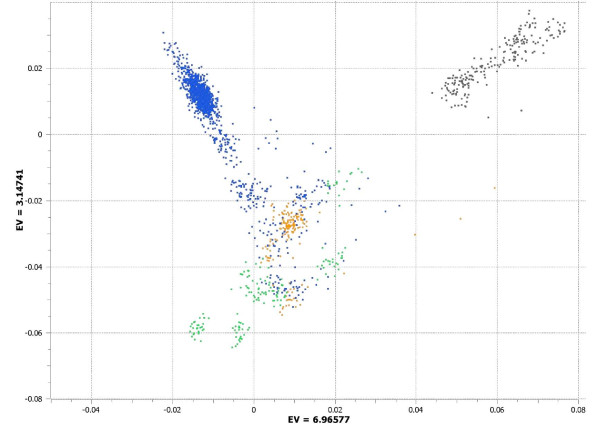
**Principal components analysis (PC1 versus PC3) of 1,312 Ashkenazi Jewish (AJ) subjects combined with Europeans (EU), Middle Easterners (ME), and Central/South Asians (CSA)**. The x-axis represents the eigenvalue (EV) for principal component 1 (PC1) and the y-axis represents the eigenvalue for principal component 3 (PC3). Blue represents AJ, green represents EU, orange represents ME and black represents CSA.

Next, we performed principal components analysis (PCA) on the full sample of AJ individuals and the neighboring HGDP populations (EU, ME, and CSA). Seven significant (eigenvalue > 1) principal components emerged, with the first two principal components (Additional file 4) centering the AJ population at the vertex of two diagonals defined by EU and ME populations, consistent with prior reports [23,24]. However, the third principal component (PC3; eigenvalue = 3.14741) differentiated the AJ population from all others (Figure 3). Note that AJ are not intermediate to EU and ME on this factor; rather, AJ are located on a dimension orthogonal to the primary component (PC1) defining these other two populations. Comparison with ADMIXTURE results demonstrated that PC3 was capturing the same variance detected by maximum likelihood methods. Across AJ and all neighboring HGDP populations, the score derived from ADMIXTURE cluster 3 (that is, amount of pea green per individual in Figure 1) was strongly correlated (r = 0.81) with PC3 score. Within AJ alone, correlation was nearly perfect (r = 0.99), indicating a strong convergence across these two methods.

**Figure 4:**
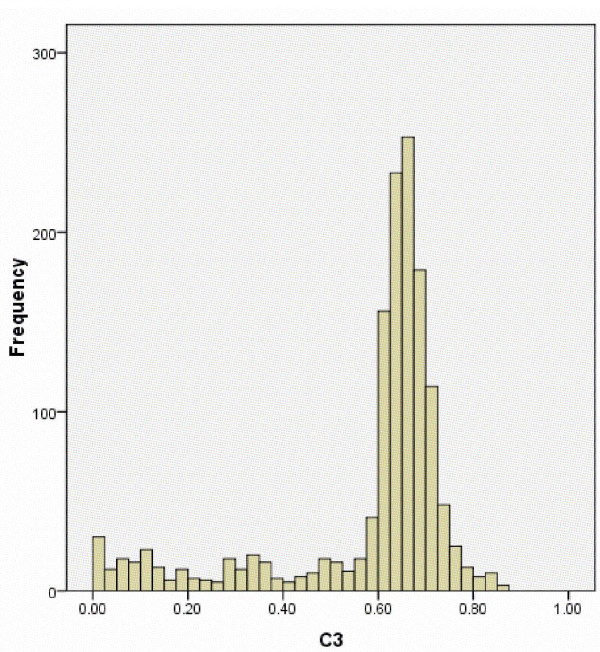
**Histogram distribution of cluster 3 (C3) scores of Ashkenazi Jewish (AJ) individuals derived from ADMIXTURE analysis**. The x-axis represents C3 scores and the y-axis represents the frequency.

**Figure 5:**
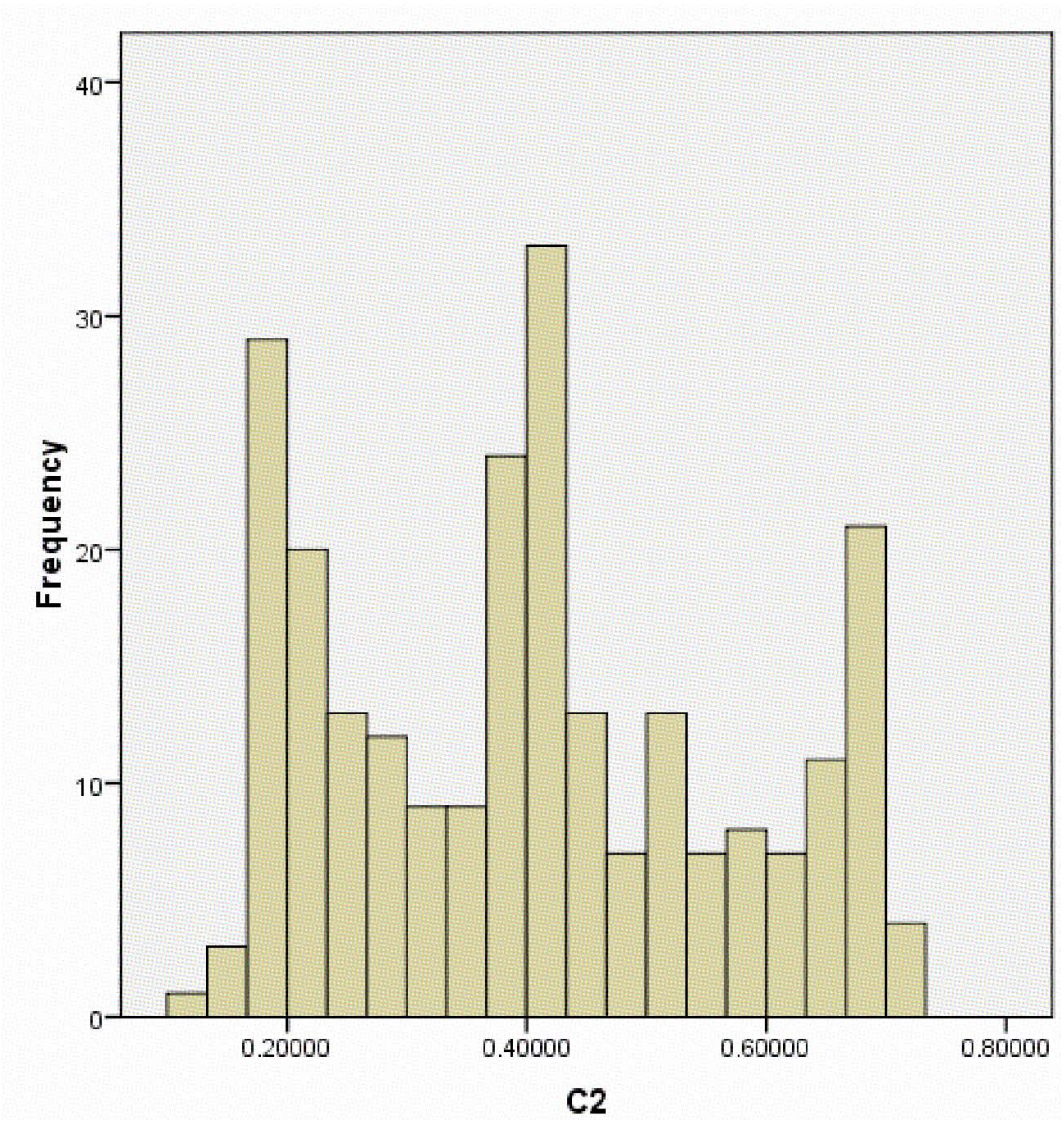
**Histogram distribution of cluster 2 (C2) scores of Ashkenazi Jewish (AJ) individuals derived from ADMIXTURE analysis**. The histogram distribution includes only those AJ subjects with low scores (< 0.475) on the Ashkenazi-specific cluster score (C3). The x-axis represents C2 scores and the y-axis represents the frequency.

While the large majority of AJ subjects cluster tightly together, it is apparent in Figure 3 that a subset of self-described AJ individuals approach or overlap the EU and ME cluster, similar to what was observed in the ADMIXTURE analysis (Figure 1). Examination of a frequency histogram for ADMIXTURE cluster 3 (C3) scores (essentially identical to PC3 scores for AJ subjects) demonstrates that 75% of AJ subjects met a strict cutoff for C3 score > 0.6 (Figure 4). More broadly, 83% fall between 0.475 and 0.875, centering on the peak observed on the histogram at C3 ≈ 0.675. Notably, as shown in Figure 5, all subjects with C3 < 0.475 fall within three peaks on C2, which is the ADMIXTURE cluster score representing the EU ancestral population (red in Figure 1). These presumably represent individuals with one, two, or three non-Ashkenazi (European or Middle Eastern) grandparents, notwithstanding their self-report of four Ashkenazi grandparents.

### Genetic distances between populations

The analyses described above demonstrate a dimension of allelic variation that is specific to the AJ cohort. Nevertheless, the second principal component (PC2) of the PCA places AJ subjects intermediate to EU and ME populations; therefore, we further tested the position of the AJ population relative to these neighboring populations using standard tests of genetic distance (F_ST_). Consistent with prior literature [23,24], pairwise genetic distances were somewhat smaller between AJ and EU populations than between AJ and ME populations, although all pairwise differences were statistically significant for AJ (Table 1). Surprisingly, however, AJ-EU distances were slightly larger than distances between Palestinian and EU samples, for each EU cohort (except Russian). For example, while both AJ and Palestinian cohorts showed the smallest F_ST _value in comparison with the Tuscan group, the Palestinian-Tuscan difference was not statistically significant (F_ST _= 0.0079), whereas the AJ-Tuscan distance was nearly twice as large (F_ST _= 0.013).

**Table 1:** Pairwise genetic distances (F_st_) between Ashkenazi Jewish, European and Middle Eastern populations

		Europe								Middle East			
			
Population	Ashkenazi	Basque	French	Sardinian	Tuscan	Italian	Orcadian	Adygei	Russian	Mozabite	Druze	Palestinian	Bedouin
Ashkenazi													
Basque	**0.0216**												
French	**0.0147**	**0.0069**											
Sardinian	**0.0206**	**0.0127**	**0.0087**										
Tuscan	**0.013**	0.0097	0.0024	0.0072									
Italian	**0.0136**	0.0079	0.0012	0.0064	0.0014								
Orcadian	**0.0197**	**0.0114**	0.0041	**0.0154**	0.008	0.0074							
Adygei	**0.0146**	**0.0176**	**0.009**	**0.0181**	0.0078	**0.0086**	**0.0137**						
Russian	**0.0172**	**0.0134**	**0.0054**	**0.019**	0.0094	0.0086	**0.0072**	**0.0119**					
Mozabite	**0.029**	**0.037**	**0.0307**	**0.0318**	**0.0258**	**0.0272**	**0.0376**	**0.0316**	**0.0368**				
Druze	**0.0173**	**0.0206**	**0.0136**	**0.0166**	0.009	**0.0108**	**0.02**	**0.0121**	**0.0205**	**0.0277**			
Palestinian	**0.0155**	**0.0202**	**0.0134**	**0.0157**	0.0079	**0.0099**	**0.0193**	**0.0113**	**0.0195**	**0.0209**	**0.0092**		
Bedouin	**0.0189**	**0.0253**	**0.0185**	**0.0204**	0.012	**0.0147**	**0.0247**	**0.0161**	**0.0252**	**0.0208**	**0.0123**	**0.0077**	

Bold indicates significance, *P *< 0.05, Fisher's exact test, 10,000 permutations.

### Residual intra-population structure

**Figure 6:**
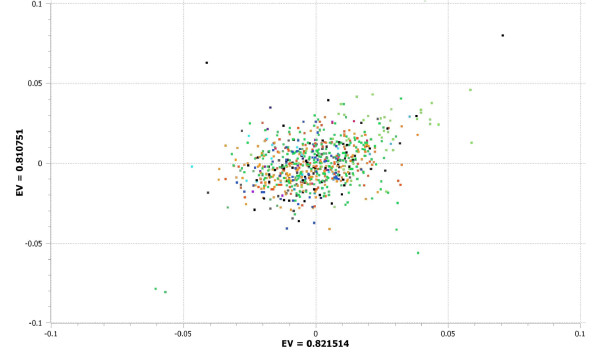
**Intra-population principal components analysis of 1,312 Ashkenazi Jewish (AJ) individuals with cluster 3 (C3) scores > 0.6 derived from ADMIXTURE analysis**. The x-axis represents the eigenvalue (EV) for principal component 1 (PC1) and the y-axis represents the eigenvalue for principal component 2 (PC2). Different colors represent different geographical origins of AJ individuals.

We next sought to examine whether residual structure could be detected in the subgroup of AJ subjects without clear evidence of European admixture. We tested the subsamples defined by both the strict (C3 > 0.6) and broad (C3 ≥0.475) cutoffs described above. As shown in Figure 6, the strict cutoff results in a PCA with no evidence of significant structure. All eigenvalues are substantially < 1 (each of the top two PCs had eigenvalues of approximately 0.8), and the plot of PC1 versus PC2 is roughly circular and demonstrates no relationship to country of origin for AJ subjects. Using the broader cutoff, a modest but non-negligible degree of structure becomes apparent (Additional file 5). As can be seen in Additional file 5, however, there is still no apparent geographic correlate to the first two PCs.

### Implications for health and disease

Next, we sought to identify which genetic variants were contributing to the AJ-specific ancestry factor identified in Figure 1. Allelic contributions to the ADMIXTURE-based cluster 3 (C3) scores were examined using quantitative GWAS (additive model comparing C3 against approximately 739 K high-quality SNPs) in all (*n *= 1,312 after all quality control procedures) AJ samples. A total of approximately 13,841 SNPs were strongly (*P *< 10^-6^) associated with C3 (Additional file 6).

Biological pathway analysis using the ALIGATOR program (details in Materials and methods) indicated that our GWAS of C3 scores yielded genes that were significantly over-represented (*P *< 0.001) in ten Gene Ontology (GO) categories (Table 2). Only approximately three such categories would be expected in a random study with similar parameters, yielding a study-wide significant *P*-value (*P *= 0.0248); this empirically determined *P*-value (based on permutation of 10,000 simulated studies of equal gene set length) indicates that results are significantly greater than would be obtained by chance.

**Table 2:** Gene Ontology categories significantly over-represented (*P *< 0.001) in ALIGATOR analysis

GO category	GO type	Genes in category	Genes on list	Expected on list	*P*-value	Expected hits per study	Function
GO: 0031667	Process	89	22	9.69	0	0.06	Response to nutrient levels
GO: 0007584	Process	55	18	6.04	0	0.06	Response to nutrient
GO: 0005829	Cellular	856	135	98.43	0.00002	0.1	Cytosol
GO: 0051059	Function	22	10	2.37	0.00004	0.15	NF-kappaB binding
GO: 0009991	Process	100	23	10.42	0.00006	0.19	Response to extracellular stimulus
GO: 0030321	Process	6	5	0.94	0.00028	0.7	Transepithelial chloride transport
GO: 0006414	Process	83	10	2.91	0.00036	0.88	Translational elongation
GO: 0050957	Process	8	6	2.71	0.0007	1.69	Equilibrioception
GO: 0030127	Cellular	7	4	0.55	0.00086	2.07	COPII vesicle coat
GO: 0012507	Cellular	7	4	0.55	0.00086	2.07	ER to Golgi vesicle membrane

Intriguingly, several of the statistically significant GO process categories in Table 2 include autosomal recessive disease-causing genes marked by relatively high-frequency Ashkenazi-specific mutations. For example, five of the six genes involved in transepithelial chloride transport (GO:0030321) are significantly associated with C3 scores; these include *CFTR*, a gene that harbors characteristic mutations that cause the increased prevalence of cystic fibrosis in the Ashkenazi population [26]. Similarly, six out of eight genes involved in equilibrioception (GO:0050597) are on the C3 GWAS list, including *PCDH15 *and *CLRN1*. Specific founder mutations in these two genes are responsible for increased prevalence of Usher syndrome (types I and III) in the Ashkenazi population [27,28]. Notably, both of these GO categories also were significant in a complementary gene-set enrichment analysis using GSA-SNP (details in Materials and methods). In the GSA-SNP analysis, the equilibrioception category demonstrated enrichment at nearly four standard deviations beyond the mean of all GO categories (Z = 3.99, *P *= 3.37E-05, false discovery rate (FDR) = 0.002); transepithelial chloride transport was also enriched more than 3 standard deviations beyond the mean (Z = 3.09; *P *= 9.98E-04; FDR = 0.029). However, the other categories listed in Table 2 did not achieve corrected significance levels (FDR > 0.05, *P *> 0.002) on the GSA-SNP enrichment list.

Of the SNPs crossing the threshold boundary (*P *< 10^-6^) for association to C3 scores, 417 were located in the coding region of more than 300 genes. Table 3 lists 15 of these that have been functionally characterized by research cited in PubMed (the full list is available in Additional file 2). Minor allele frequency in the AJ cohort is listed for each of these SNPs; for comparison, minor allele frequencies for the three 'Caucasian' non-Hispanic HapMap populations (representing northern Europe (CEU), southern Europe (TSI), and India (GIH)), are also presented in Table 3. For example, it can be seen that the A allele at rs213950, coding for the Met variant at position 470 in *CFTR*, is significantly under-represented in the AJ population compared to the other three (and all other HapMap populations, as well). Given that most mutations causing cystic fibrosis are found on M470 haplotypes [29,30], this could represent the effects of purifying selection. Two additional coding variants in genes associated with recessive AJ diseases (*HEXA *and *IKBKAP*) are also detected in this analysis.

**Table 3:** Fifteen coding variants with functionally characterized SNPs crossing the threshold (*P *< 10^-6^) for association with C3 scores

Gene	Marker	Chromosome	Chromosome position	Amino acid position	Amino acid change	Correlation/trend *P*	Correlation/trend R	Minor allele	AJ frequency	CEU frequency	TSI frequency	GIH frequency
*CFTR*	rs213950	7	116986769	1408	V | M	2.29E-08	-0.155	A	0.287	0.487	0.347	0.489
*ABCB1*	rs1045642	7	86976581	3435		4.95E-11	-0.183	A	0.367	0.571	0.466	0.597
*GSTP1*	rs1138272	11	67110155	341	A | V	4.74E-08	-0.152	T	0.046	0.097	0.062	0.091
*MTHFR*	rs1801133	1	11778965	665	A | V	6.70E-10	0.172	A	0.435	0.31	0.46	0.17
*PPARG*	rs1801282	3	12368125	34	P | A	3.21E-09	-0.165	G	0.058	0.097	0.074	0.091
*MC1R*	rs1805005	16	88513345	178	V | L	4.80E-16	0.227	T	0.266	0.006	0.159	0.006
*PLAU*	rs2227564	10	75343107	422	L | P	4.82E-08	-0.152	T	0.107	0.239	0.188	0.369
*PER1*	rs2253820	17	7988894	2361		1.22E-11	0.188	T	0.378	0.185	0.165	0.222
*ALF1*	rs2269475	6	31691910	43	R | W	2.70E-08	-0.155	T	0.039	0.159	0.091	0.097
*HLA-DRA*	rs3135391	6	32518965	402		8.47E-10	-0.171	A	0.034	0.19	0.08	0.017
*SH2B3*	rs3184504	12	110368991	784	W | R	1.36E-10	-0.179	C	0.365	0.555	0.472	0.869
*DKK3*	rs3206824	11	11942637	1003	R | G	1.44E-08	-0.158	T	0.128	0.243	0.165	0.148
*LCT*	rs3754689	2	136307216	655	V | I	1.05E-09	0.17	T	0.466	0.102	0.402	0.29
*PPARGC1A*	rs3755863	4	23424620	1584		4.99E-08	0.052	T	0.473	0.412	0.511	0.398
*NOD2*	rs2067085	16	49291359	639		9.54E-08	0.151	G	0.536	0.381	0.455	0.207

AJ, Ashkenazi Jew; CEU, Northern Europe; GIH, Gujarat India; TSI, Southern Europe Tuscan.

The relative over-representation of the minor allele at rs1801133 (also known as *MTHFR *C677T) in AJ populations has been previously noted [31]; homozygosity at this allele is associated with hyperhomocysteinemia. Several novel findings are also apparent from Table 3, with potential impact on disease risk within the AJ population. For example, *SH2B3 *regulates cytokine activity, and rs3184504 within this gene has been replicably associated with risk for type 1 diabetes and celiac disease [32,33]. The AJ population has a lower frequency of the protective C allele (that is, a higher frequency of the disease-associated T allele) than any other HapMap population. Similarly, the AJ cohort has a reduced frequency of the Ala12 variant (G allele at rs1801282) in the *PPARG *gene; the Ala12 variant, while rare in all populations, reduces risk for type 2 diabetes by a factor of 0.86 [34]. The V60L variant at *MC1R*, also quite common in the AJ cohort, has been associated with melanoma in some, but not all, populations [35]. By contrast, the AJ population has a reduced frequency of the T allele at rs2227564, which has been associated with Alzheimer's disease [36]. Similarly, the AJ population has a reduced frequency of the R15W variant of the *AIF1 *gene, which has been strongly (odds ratio > 2) associated with rheumatoid arthritis [37].

We also performed a GWAS on scores derived from PC1 of the intra-population PCA depicted in Additional file 5. As shown in Additional file 7, this source of population variance was strictly accounted for by allelic differences in the major histocompatibility complex (MHC). Notably, the MHC alleles associated with intra-AJ population structure are completely different from the MHC component associated with the inter-population analysis (Additional file 6). For example, the AJ population is differentiated from neighboring non-AJ populations by a reduced frequency of the A allele at rs3135391 (Table 3), which tags the HLA-DRB*1501 allele. This allele has been associated with susceptibility to multiple sclerosis and other autoimmune diseases [38]. By contrast, the intra-population principal component (PC1) is most strongly correlated with alleles in the class I region of the MHC - for example, rs9260952 in the region of HLA-A (*P *= 2.46 × 10^-106^) and rs3828875 (*P *= 4.76 × 10^-106^), which has been correlated with HLA-B *6701 and *3802 alleles [39].

### Ancestry informative markers

Finally, we sought to validate a set of AIMs, derived from our AJ-specific ADMIXTURE component (C3), in an independent dataset. We selected a publicly available dataset in which previous work has been published on identification of AJ-specific allelic variation [21]. Of the 13,841 SNPs that strongly (*P *< 10^-6^) correlated with C3 in our GWAS analysis, 1,357 were available in the Need *et al. *dataset [21]. PCA of these 1,357 SNPs revealed a first principal component that strongly differentiated AJ from non-AJ subjects; this result closely paralleled results of the PCA reported by Need *et al. *[21] using more than 120 K SNPs (Additional file 8 of this study versus Figure 2 in Need *et al. *[21]). As shown in Table 4, classification of subjects based on the PCA of only 1,357 SNPs derived from our ADMIXTURE analysis (Table 4) was essentially no different in classification concordance to self-reported ancestry from the original values reported by Need *et al. *[21] (Table 5).

**Table 4:** Classification of AJ individuals derived from PCA clustering using 1,357 SNPs obtained from ADMIXTURE analysis

		Ashkenazi Jewish grandparents (self-report)					
		
		0	1	2	3	4	Total
Predicted AJ?	N	501	5	3	0	0	509
	Y	6	3	34	4	55	102
Total		507	8	37	4	55	611

**Table 5:** Classification of AJ individuals derived from PCA clustering using 121,834 SNPs in Need *et al. *[21]

		Ashkenazi Jewish grandparents (self-report)					
		
		0	1	2	3	4	Total
Predicted AJ?	N	496	7	2	0	0	507
	Y	11	1	35	4	55	104
Total		507	8	37	4	55	611

Selecting only the 103 SNPs that strongly (*P *< 10^-6^) loaded onto the first principal component from this analysis yielded results that were virtually indistinguishable (Table 6). As a final validation, we then applied these 103 AIMs to compare the original (*n *= 1,312) AJ individuals used in this study to the HGDP European cohort in order to evaluate the power to identify AJ individuals with putative European admixture (that is, one or more non-AJ grandparents). Of the 103 AIMS, 89 were available in the HGDP European individuals' dataset. The PCA result clearly illustrates the separation of full AJ individuals (C3 admixture score > 0.475) from non-full AJ individuals (C3 admixture score < 0.475) overlapping with European individuals (Additional file 9). The identified non-full AJ individuals account for 16.9% of the total original AJ individuals, which is in high concordance with our previous finding using ADMIXTURE analysis. This list of 103 SNPs (Additional file 2), the smallest such set for the AJ population to date, therefore provides a robust set of AIMs for identifying sub-cohorts with AJ heritage within the context of disease-mapping studies examining European or European-American populations. For studies in which cost of additional SNPs is not a factor, please see the full list of PCA-derived SNPs in Additional file 2.

**Table 6:** Classification of AJ individuals derived from PCA clustering using AIM 103 SNPs obtained from ADMIXTURE analysis

		Ashkenazi Jewish grandparents (self-report)					
		
		0	1	2	3	4	Total
Predicted AJ?	N	499	5	6	0	0	510
	Y	8	3	31	4	55	101
Total		507	8	37	4	55	611

## Discussion

While there have been several population genetics studies of Jewish cohorts published in the past two years [21-25], the findings of the present study are novel in several ways. First, prior studies have emphasized commonalities amongst Jewish sub-populations, as well as relative proximity to European and Levantine populations. By contrast, the present study took the complementary approach of defining the spectrum of autosomal variation that is AJ-specific. Moreover, using novel pathway analyses, the present study related population genetic variation to patterns of disease propensity in the Ashkenazi population. Second, the present study examined intra-Ashkenazi variation. Finally, we provide a robust yet compact list of AIMs for the Ashkenazi population.

The primary result of the present study is the specification of the allelic content of an autosomal genetic signature that can distinguish the Ashkenazi Jewish population from both its host populations in Europe and other populations that originate in the same geographic area of the Levant. To our knowledge, ours is the first study of the Ashkenazi population to utilize cross-validation metrics to identify the optimal solution to the assignment of population ancestry scores. Previous studies using similar approaches have demonstrated the ability of genomic information to differentiate Ashkenazi samples from those drawn from other populations [1,20-25]. However, each of these studies has suggested that AJ samples represent an intermediate position or admixture between European and Levantine populations. Although one recent paper suggested 30 to 60% European admixture in Ashkenazi and other Jewish samples [24], the present study found relatively little (≤10%) overlap of AJ genetic ancestry components in non-AJ Levantine populations. In the statistically optimal ADMIXTURE result in our study, European admixture followed a pattern indicative of second-generation admixture rather than deeper mingling with the host populations. Moreover, pairwise genetic distances were not consistent with an intermediate positioning of the AJ population relative to the European and Levantine populations.

It should be emphasized that these results do not suggest an independent (for example, Khazar or non-Levantine) lineage for the AJ population, a hypothesis that has generally been ruled out by prior literature [16,17,24]. Rather, Table 1 demonstrates relative proximity amongst several populations with Mediterranean heritage, including the AJ, Palestinians, and Italians, suggestive of an ancient common deme. Additionally, the F_ST _data indicate approximately equal genetic distances between the AJ and western (French), eastern (Adygei), and Middle Eastern (Palestinian) cohorts, consistent with the suggestion that founder effects and subsequent drift account for the data more strongly than substantial local in-mixture with the European host populations in the last 1,000 years.

Moreover, the present study is the first to examine residual intra-population variance in AJ samples in comparison to host European populations. Results of our intra-AJ principal components analysis indicated that residual structure was minimal, was not related to geographic origin within Europe, and did not map onto differences in host population. Taken together, these data most likely reflect the unique contributions of the AJ founder population to the genetic make-up of present-day Ashkenazim. At the same time, it is acknowledged that our autosomal data may not capture certain components of ancestry that are accessible to mitochondrial DNA and Y-chromosome studies, such as sex differences in origin and number of founders [16-18].

Having identified this AJ-specific signature, we then sought to characterize its primary allelic content in order to determine potential relevance to future disease mapping studies. We developed a robust yet compact set of AIMs that can be applied to refine studies of European or European-American cohorts, which are still the most commonly used in disease mapping GWASs. These AIMs will also be useful in future GWASs of AJ cohorts, insofar as they can identify individuals with varying degrees of recent European admixture, thereby reducing residual intra-population structure (Figure 6). The lack of significant intra-population structure suggests that the AJ population may be useful for disease-mapping studies, with the possibility of enhanced signal-to-noise for the detection of (at least a subset) of disease-related alleles [15].

Alleles within the MHC were the most substantial contributors to both inter-population and intra-population variance. MHC markers comprised approximately 6% of all approximately 13,841 SNPs that were correlated with the AJ-specific signature, including polymorphisms in both class I and class II genes. Prior research has consistently demonstrated the MHC to be most sensitive to population differences [40], typically due to geographic differences in exposure history [41]. These population differences have implications for susceptibility to autoimmune diseases [42], and may account for the increased rate of pemphigus vulgaris in AJ individuals [43]. Recent studies associating SNPs in the MHC with serious drug-induced side effects [44], viral load in HIV [45] and psychiatric illness [46] also indicate the clinical relevance of more extensive elaboration of population differences in MHC alleles.

Characterization of the AJ-specific component also resulted in the identification of several coding variants known to be associated with disease, and was able to detect markers in *CFTR *and *NOD2 *that are relevant to increased prevalence of cystic fibrosis and Crohn's disease in the Ashkenazi population [47,48]. Perhaps the most surprising result from the present study, however, was the over-representation of GO categories containing disease-bearing genes commonly associated with the AJ population. For example, the AJ cohort did not merely differ from other populations in *CFTR *allele frequencies, but also in allelic frequencies in most other genes associated with transepithelial chloride transport. However, it should be noted that these data do not provide specific evidence of causality between the existence of AJ-prevalent disease-causing mutations in these pathways and the over-representation of certain common alleles in related genes. Speculatively, these results suggest the possibility that deleterious recessive alleles may persist at relatively high frequencies in the AJ population due to epistatic effects with other genes in the same biological pathway, which also display altered allelic frequencies in the AJ population.

## Conclusions

The present study characterized statistically significant components of autosomal variation specific to the AJ population. By focusing on common variants available on a dense GWAS platform, results add to prior literature on rare, disease-causing mutations that are over-represented in the Ashkenazi population. GO analysis points to significant allele frequency differences in multiple genes in pathways implicated by AJ-associated diseases such as cystic fibrosis and Usher's syndrome. However, it will be important for future research to determine which elements of this genetic signature are shared with non-AJ populations, and may therefore be reflective of ancient founder effects, as opposed to more recent founder effects specific to the introduction and expansion of the Jewish people into Europe.

## Materials and methods

### Samples

The AJ cohort consisted of 1,394 volunteers (986 male, 408 female) recruited from the Israeli blood bank. Each subject self-reported that all four grandparents were of AJ origin, and all subjects provided written, informed consent. Subsequent to genomic DNA extraction from blood samples through use of the Nucleon kit (Pharmacia, Piscataway, NJ, USA), all samples were fully anonymized prior to genotyping and analysis, under protocols approved by the National Genetic Committee of the Ministry of Health (Israel) and the Institutional Review Board of the North Shore-LIJ Health System.

The HGDP genome-wide genotype data containing 1,043 individuals from 51 worldwide population groups were obtained from the HGDP database [49]. The sample sizes for many individual groups were very small and grouped together based on their geographical distribution and ethnicity for comparison analysis as suggested [50].

Additional genotype data on 611 Caucasian subjects recruited at Duke University, including 94 individuals who self-reported having one or more AJ grandparents, were from Need *at al. *[21].

### Genotyping and quality control

Genotyping of AJ samples was performed using Illumina HumanOmni1-Quad arrays according to the manufacturer's specifications. The samples were subjected for genotyping quality control filters, for example, samples call rate > 97%, SNP call rate > 98%, Hardy-Weinberg exact test *P *< 0.000001. The resulting individuals were tested for gender mismatch based on X chromosome genotype using Sex check estimation at PLINK (v1.07) [51]. Cryptic identity and first-degree relatedness within individuals were examined using pairwise IBD estimation in PLINK performed on 128 K LD (linkage disequilibrium) pruned (r2 > 0.2) genomewide SNPs; one individual in each pair was randomly excluded. The final dataset contains 1,312 individuals with 739,409 SNPs with 99.86% average call rates.

The HGDP samples were genotyped on the Illumina HumanHap 650 k bedchip as previously described and filtered based on a sample call rate > 98.5%, resulting in 1,043 individuals with 660,918 SNPs. This dataset was again filtered based on a SNP call rate > 95%. The filtered AJ dataset was merged with the HGDP dataset and the resulting merged dataset contained 281,232 SNPs common to the two cohorts with an average call rate of 99.5%.

To perform inter-population comparison analysis (for example, ancestry estimation and PCA) the AJ and HGDP merged dataset was pruned using a LD threshold of r^2 ^> 0.2 at PLINK (v1.07). The resulting dataset contained 95,600 unlinked genomewide SNPs shared by the AJ and HGDP samples with an average call rate of 99.7%.

Genotyping of the Duke samples was performed on Illumina Infinium HumanHap550 version 1, version 3 and 610-quad chips. The dataset contains information on 121,834 LD-pruned (r^2 ^> 0.3) SNPs and was used to validate an AIM panel specific to AJ ancestry.

### Ancestry estimation

The population structure analysis was performed using the maximum likelihood based ADMIXTURE program [52]. The maximum likelihood approaches are as accurate as Bayesian-based estimations while being computationally tractable with genomewide markers within a reasonable time. This algorithm is also considered to be more accurate and faster than the expectation-maximization-based program FRAPPE [53].

To detect underlying ancestral population clustering, AJ samples were compared with members of three neighboring population groups derived from the HGDP: EU (*n *= 159), ME (*n *= 163), and CAS (*n *= 177, excluding Kalash as per Behar *et al. *[23]). We performed ancestry estimation by randomly selecting *n *= 175 AJ subjects of varying national origins, in order to maintain a roughly equal sample size with each of the other three HGDP groups. Briefly, the ADMIXTURE algorithm models the genomic data from each subject as a combination of K ancestral populations, where K can be any number ≥2. ADMIXTURE output results were systematically plotted using the Distruct program [54], which permits visual determination of similarities and differences in ancestral make-up of each population. More formally, ten-fold cross-validation 'C' scores were computed for each K separately to determine the best fit model for ancestry estimation. We then re-performed ADMIXTURE and cross-validation analyses ten times to develop statistical confidence intervals around fit scores for each of the ancestry estimates. In order to test the effect of varying sample sizes on the analyses, and to exploit the full sample size of AJ individuals available, these analyses were repeated using *n *= 350, *n *= 700, *n *= 1,050, and all *n *= 1,312 AJ samples. The ancestry estimation was also performed with all HGDP groups using both randomly selected *n *= 175 AJ and all *n *= 1,312 AJ individuals. This analysis was carried out with 95,600 LD-pruned unlinked SNPs for K = 2 to 20, where K is the prior assumption of theoretical ancestral population.

### Principal component analysis

PCA was performed to examine the inter- and intra-population distribution using EIGENSTRAT [55] as implemented in SNP & Variation Suite v7.3 (Golden Helix, Bozeman, MT, USA). The previously described 95,600 LD-pruned unlinked SNPs were used to perform inter-population PCA with a randomly selected subset of *n *= 175 AJ samples with members of the three neighboring population groups used in the ADMIXTURE analysis. Intra-population PCA was performed for all AJ individuals who clustered strongly with the AJ cohort (based on admixture analysis), using all 739,409 high quality SNPs.

### Calculation of distances between populations

Pairwise F_ST _values for all pairs of populations were estimated using GENEPOP v4.1 [56] by a weighted analysis of variance [57]. For each locus, an unbiased estimate of the *P*-value was also computed using Fisher's exact probability test, and the significance of each pairwise distance was empirically tested using a permutation algorithm (*n *= 5,000 runs) as previously described [58].

### Quantitative genome-wide association study

To identify which genetic variants were contributing to the AJ-specific ancestry dimension, a quantitative GWAS was performed based on ADMIXTURE-derived AJ-specific cluster scores for all AJ samples (*n *= 1,312) with 739,409 high quality SNPs. A quantitative GWAS was also performed on scores derived from PC1 of the intra-population PCA to identify the source of this genetic variation.

### Gene Ontology enrichment analysis

To determine whether any biologically relevant pathways were over-represented amongst this list of associated SNPs, we utilized Association LIst Go AnnoTatOR (ALIGATOR) [59]. Like the Database for Annotation, Visualization, and Integrated Discovery (DAVID) [60], ALIGATOR characterizes lists of genes with respect to their relative inclusion of the various GO categories. However, ALIGATOR is specifically designed for analysis of SNP data (as opposed to gene expression data), controlling for the size of each gene and the number of SNPs present on the array.

After assigning each SNP to the closest gene, and calculating the number of genes in each GO category appearing above a specified threshold (for example, *P *< 10^-6^) in the quantitative GWAS analysis, the degree of over-representation of specific GO categories is then tested using two sets of permutations. First, the SNPs appearing above and below the GWAS cutoff are permuted (50,000 times), to determine the likelihood that a given GO category is over-represented in the list of significant SNPs. Thus, each GO category is assigned an empirically determined *P*-value. Second, simulated studies are permuted (10,000 times) in order to determine whether the number of categories designated as 'over-represented' (that is, category-specific *P*-values < 0.05, < 0.01, and < 0.001) is statistically unlikely given the number of genes on the list. Note that the initial threshold boundary (*P *< 10^-6^) is not, strictly speaking, a statistical threshold for significance. Rather, it is selected based on the assumption, intrinsic to the polygenic model approach, that true associations exist below the threshold of strict genomewide significance [61,62]. Thus, the purpose of the ontology enrichment analysis is to identify biologically relevant signals emerging from the pattern of observed associations, irrespective of strict statistical significance, and even if no SNPs achieved strict genomewide significance [63]. Following the suggestions of the software developer, the algorithm tends to be most robust when approximately 10% of all genes appear on the list (P Holmans, personal communication); consequently, we selected a threshold that resulted in 12.4% (2,349 out of 19,011 genes with GO annotations and a minimum set size of 2) of all genes submitted to ALIGATOR.

While ALIGATOR was the primary method of pathway analysis, due to its unique two-stage approach to control for study-wide significance, it is acknowledged that there are many ways to evaluate aggregation of the associated SNPs within biological pathways [60]. Consequently, we sought to validate results using the recently developed GSA-SNP program [61], which utilizes a fundamentally different approach. The essential difference between ALIGATOR and GSA-SNP is that the first method uses overrepresentation based analysis, whereas the second uses gene-set enrichment-based analysis. Overrepresentation based analysis defines significant SNPs by a pre-specified *P*-value threshold, then counts significant genes in each pathway, whereas gene-set enrichment analysis considers all the SNPs in the analysis and then ranks the gene sets in order of significance [64]. Moreover, ALIGATOR bases its analysis on the single most strongly associated SNP in each gene, whereas GSA-SNP permits the use of the *k*th (*k *= 1, 2, 3, 4 or 5) best *P*-value to represent each gene. We utilized the authors' recommended default of the second best *P*-value within each gene, which removes singleton false-positive signals and provides a more symmetric distribution to the gene scores [65]. Significant gene set enrichment was determined by the z-statistic, with FDR < 0.05 based on Benjamini-Hochberg correction.

### Ancestry informative markers

A potential set of AIMs specific to AJ was selected based on the quantitative GWAS of the AJ-specific component derived from ADMIXTURE analysis. This set of candidate AIMs was reduced and validated using a publicly available dataset previously used for identification of AJ-specific allelic variation [21]. After identification of overlapping markers, PCA was performed on the Need *et al. *dataset [21] using the candidate AIMs, and results were compared to self-reported AJ ancestry.

## Abbreviations

AIM: ancestry informative marker; AJ: Ashkenazi Jewish; ALIGATOR: Association LIst Go AnnoTatOR; CSA: Central-South Asian; EU: European; FDR: false discovery rate; GO: Gene Ontology; GWAS: genome-wide association study; HGDP: human genome diversity panel; LD: linkage disequilibrium; ME: Middle Eastern; MHC: major histocompatibility complex; PC1: first principal component; PC2: second principal component; PC3: third principal component; PCA: principal components analysis; SD: standard deviation; SNP: single nucleotide polymorphism.

## Authors' contributions

SG and TL carried out the analysis and drafted the manuscript. JR participated in the analysis. AL and PG carried out genotyping of the samples. AD provided the samples. AM, JK, IP, TL and AD conceived of the study, and participated in its design and coordination and helped to draft the manuscript. All authors have read and approved the manuscript for publication.

## Supplementary Material

Additional file 1 **K = 1 to 15 with corresponding cross validation (CV) score and standard error for 1,312 AJ individuals and HGDP individuals**. Europeans (*n *= 159), Middle Easterners (*n *= 163), and Central/South Asians (*n *= 177). Click here for file

Additional file 2 **List and annotation of 13,841 significant (*P *< 10^-6^) SNPs on Ashkenazi-specific principal component, 1,357 SNPs that were used in the initial ancestry informative marker (AIM) analysis, 103 AJ specific AIMs, 417 coding variants from 13,841 SNPs, Genetic Association Database (GAD) annotation**. Click here for file

Additional file 3 **ADMIXTURE analysis for ancestry estimation of Ashkenazi Jewish (AJ) population with seven global population groups derived from the HGDP at K = 2 through K = 14**. Each individual is represented by a thin vertical line, which is partitioned into K colored segments that represent the individual's estimated membership fractions in K clusters. Black lines separate individuals of different population groups based on geography and ethnicity. Geographical population groups are labeled below the figure. Click here for file

Additional file 4 **Principal component analysis (PC1 versus PC2) of 1,312 Ashkenazi Jewish (AJ) subjects combined with Europeans (EU), Middle Easterners (ME), and Central/South Asians (CSA)**. The x-axis represents the eigenvalue for principal component 1 (PC1) and the y-axis represents the eigenvalue for principal component 2 (PC2). Blue represents AJ, green represents EU, orange represents ME and black represents CSA. Click here for file

Additional file 5 **Intra-population principal component analysis of Ashkenazi Jewish (AJ) individuals with cluster 3 (C3) scores > 0.475 derived from ADMIXTURE analysis**. The x-axis represents the eigenvalue for principal component 1 (PC1) and the y-axis represents the eigenvalue for principal component 2 (PC2). Different colors represent different geographical origin of Ashkenazi Jewish (AJ) individuals. Click here for file

Additional file 6 **Manhattan plot for quantitative genome wide association for Ashkenazi Jewish (AJ) individuals based on cluster 3 (C3) scores derived from ADMIXTURE analysis**. The x-axis represents the chromosomes and the y-axis represents -log10 *P*-values of significance. Click here for file

Additional file 7 **Manhattan plot for quantitative genome wide association for Ashkenazi Jewish (AJ) individuals based on principal component 1 (PC1) of intra-population principal component analysis with cluster 3 (C3) scores > 0.475 derived from ADMIXTURE analysis**. The x-axis represents the chromosomes and the y-axis represents -log10 *P*-values of significance. Click here for file

Additional file 8 **Principal component analysis of Need *et al. ***[21]**cohort with designed Ashkenazi Jewish (AJ) specific ancestry informative markers**. The numbers and the corresponding colors represent the degree of self-reported Ashkenazi admixture. Click here for file

Additional file 9 **Principal component analysis of all 1,312 AJ individuals with European HGDP individuals for 89 ancestry informative markers**. Red indicates AJ individuals with C3 admixture score > 0.475, blue indicates AJ individuals with C3 admixture score < 0.475 and green indicates HGDP European individuals. Click here for file
