# Supplementary material for: Implications for health and disease in the genetic signature of the Ashkenazi Jewish population
Source: Genome Biol. 2012 Jan 25;13(1):R2. doi: 10.1186/gb-2012-13-1-r2 (PMC3334583; doi:10.1186/gb-2012-13-1-r2)
Supplement: Additional file 1 — K = 1 to 15 with corresponding cross validation (CV) score and standard error for 1,312 AJ individuals and HGDP individuals. Europeans (n = 159), Middle Easterners (n = 163), and Central/South Asians (n = 177). [file gb-2012-13-1-r2-S1.PDF]

K	CV	Standard error of CV
1	0.60960	0.00003
2	0.60797	0.00003
3	0.60728	0.00003
4	0.60662	0.00003
5	0.60638	0.00003
6	0.60630	0.00003
7	0.60623	0.00003
8	0.60622	0.00003
9	0.60623	0.00003
10	0.60627	0.00003
11	0.60633	0.00003
12	0.60632	0.00003
13	0.60643	0.00003
14	0.60646	0.00003
15	0.60658	0.00003
