# Supplementary figures and images for: Implications for health and disease in the genetic signature of the Ashkenazi Jewish population
Source: Genome Biol. 2012 Jan 25;13(1):R2. doi: 10.1186/gb-2012-13-1-r2 (PMC3334583; doi:10.1186/gb-2012-13-1-r2)

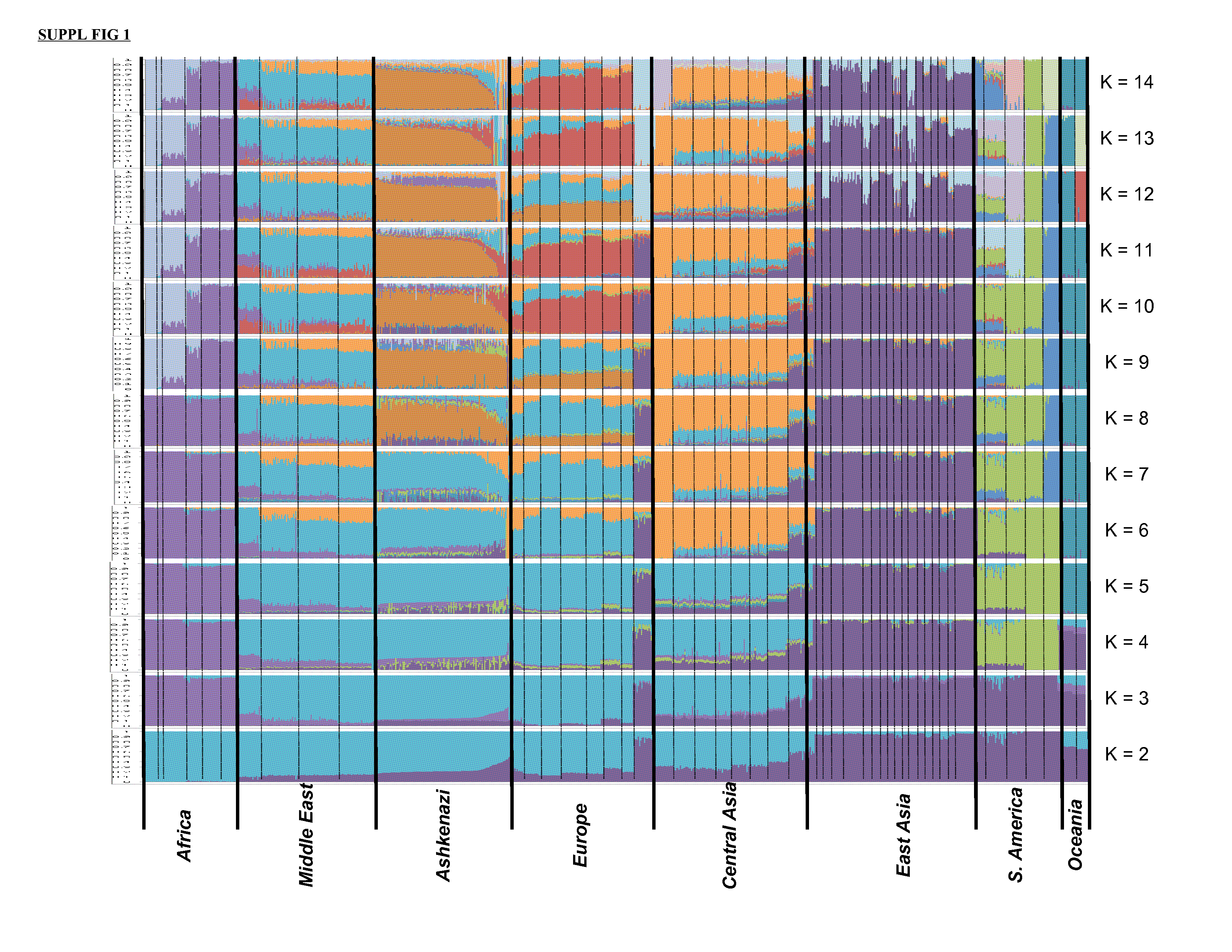

Supplement: Additional file 3 — ADMIXTURE analysis for ancestry estimation of Ashkenazi Jewish (AJ) population with seven global population groups derived from the HGDP at K = 2 through K = 14. Each individual is represented by a thin vertical line, which is partitioned into K colored segments that represent the individual's estimated membership fractions in K clusters. Black lines separate individuals of different population groups based on geography and ethnicity. Geographical population groups are labeled below the figure. [file gb-2012-13-1-r2-S3.BMP]

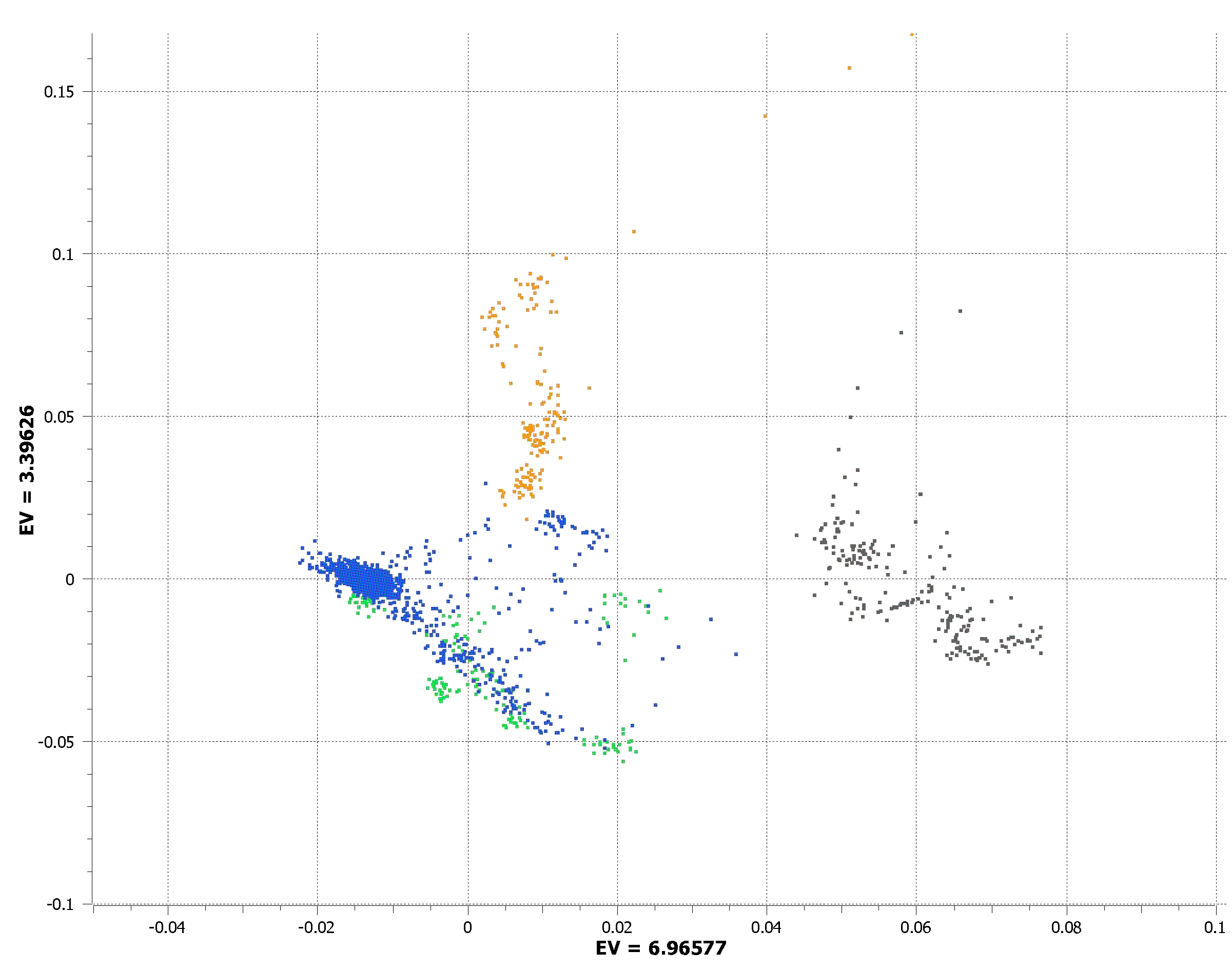

Supplement: Additional file 4 — Principal component analysis (PC1 versus PC2) of 1,312 Ashkenazi Jewish (AJ) subjects combined with Europeans (EU), Middle Easterners (ME), and Central/South Asians (CSA). The x-axis represents the eigenvalue for principal component 1 (PC1) and the y-axis represents the eigenvalue for principal component 2 (PC2). Blue represents AJ, green represents EU, orange represents ME and black represents CSA. [file gb-2012-13-1-r2-S4.BMP]

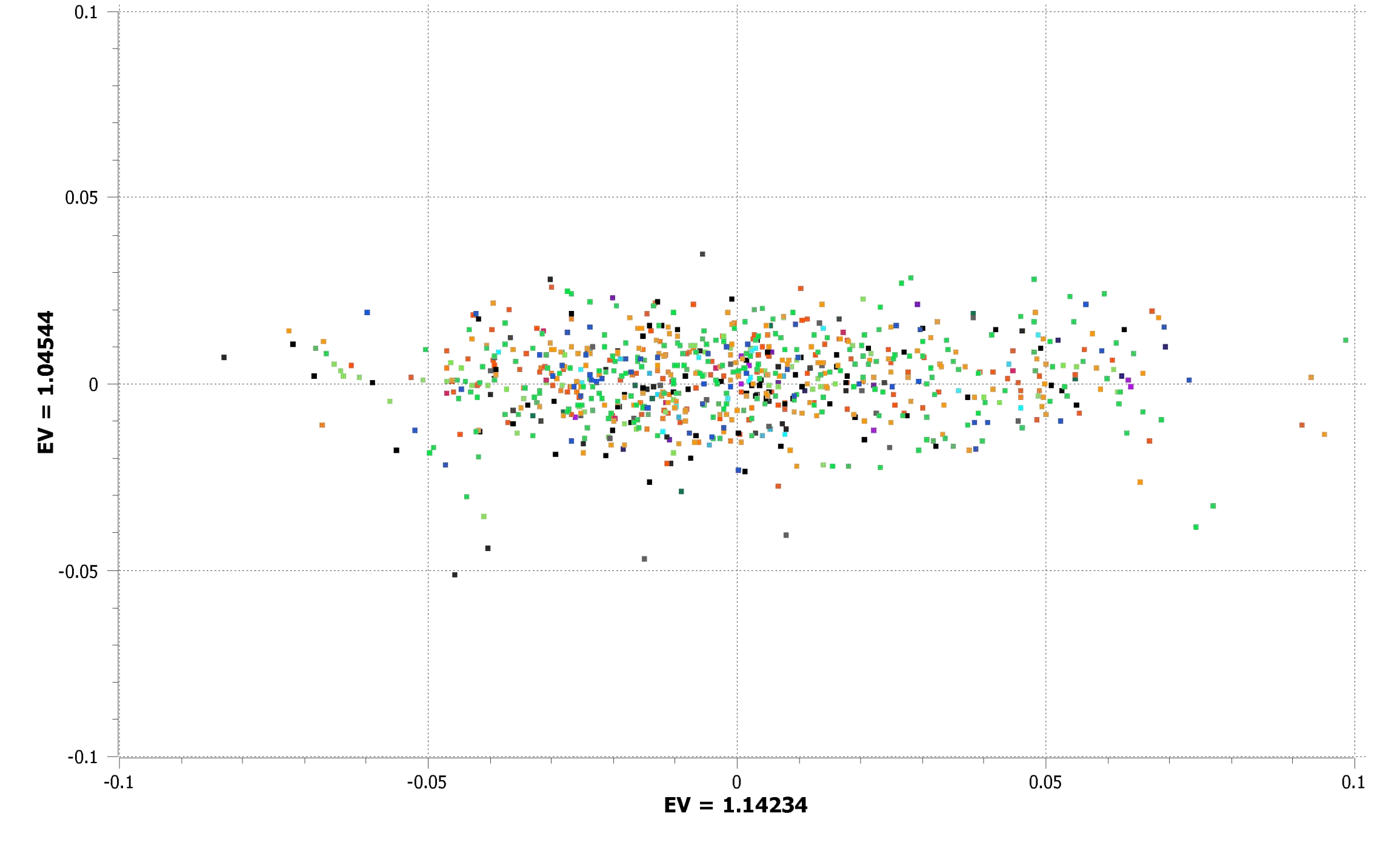

Supplement: Additional file 5 — Intra-population principal component analysis of Ashkenazi Jewish (AJ) individuals with cluster 3 (C3) scores > 0.475 derived from ADMIXTURE analysis. The x-axis represents the eigenvalue for principal component 1 (PC1) and the y-axis represents the eigenvalue for principal component 2 (PC2). Different colors represent different geographical origin of Ashkenazi Jewish (AJ) individuals. [file gb-2012-13-1-r2-S5.BMP]

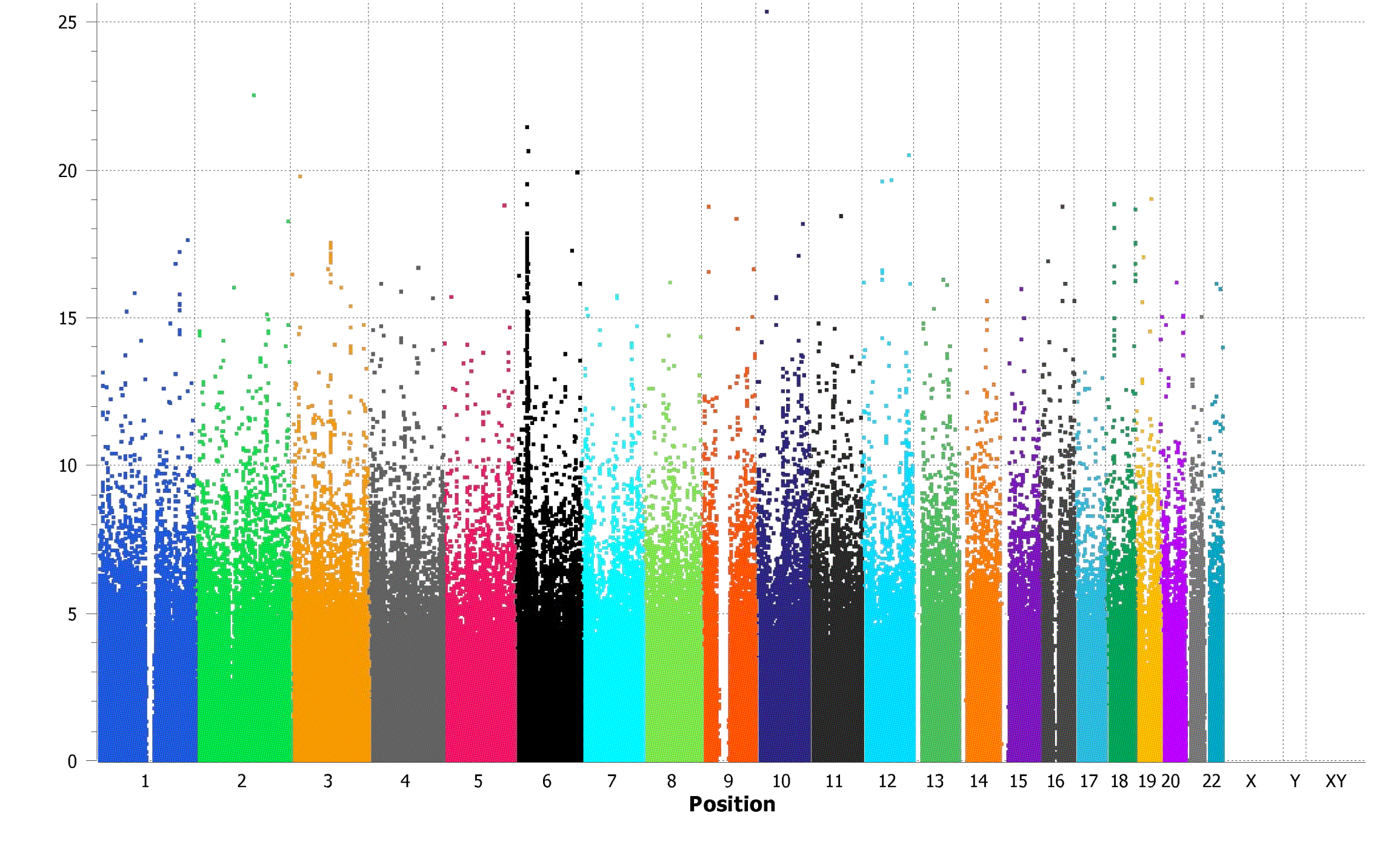

Supplement: Additional file 6 — Manhattan plot for quantitative genome wide association for Ashkenazi Jewish (AJ) individuals based on cluster 3 (C3) scores derived from ADMIXTURE analysis. The x-axis represents the chromosomes and the y-axis represents -log10 P-values of significance. [file gb-2012-13-1-r2-S6.BMP]

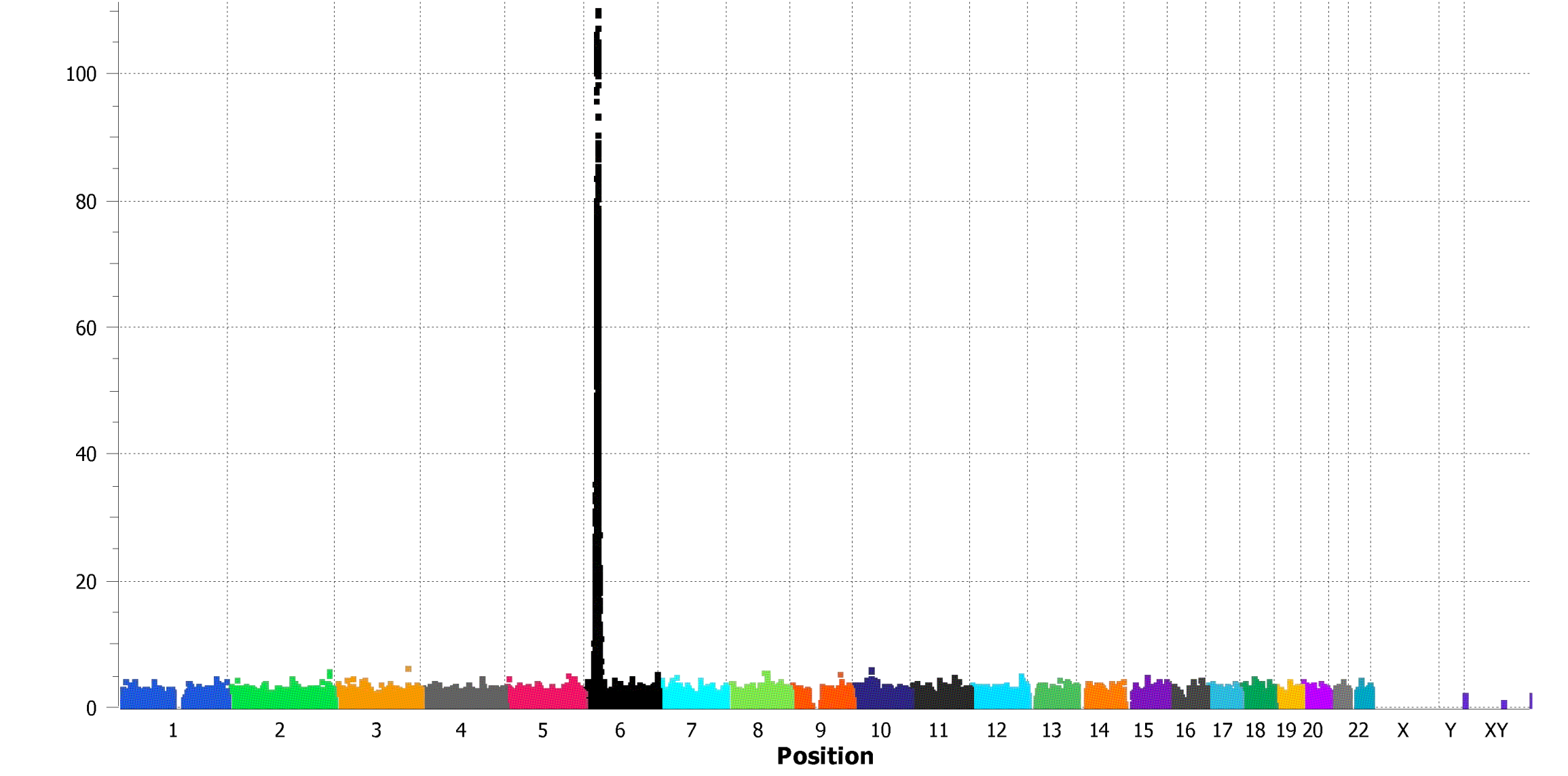

Supplement: Additional file 7 — Manhattan plot for quantitative genome wide association for Ashkenazi Jewish (AJ) individuals based on principal component 1 (PC1) of intra-population principal component analysis with cluster 3 (C3) scores > 0.475 derived from ADMIXTURE analysis. The x-axis represents the chromosomes and the y-axis represents -log10 P-values of significance. [file gb-2012-13-1-r2-S7.BMP]

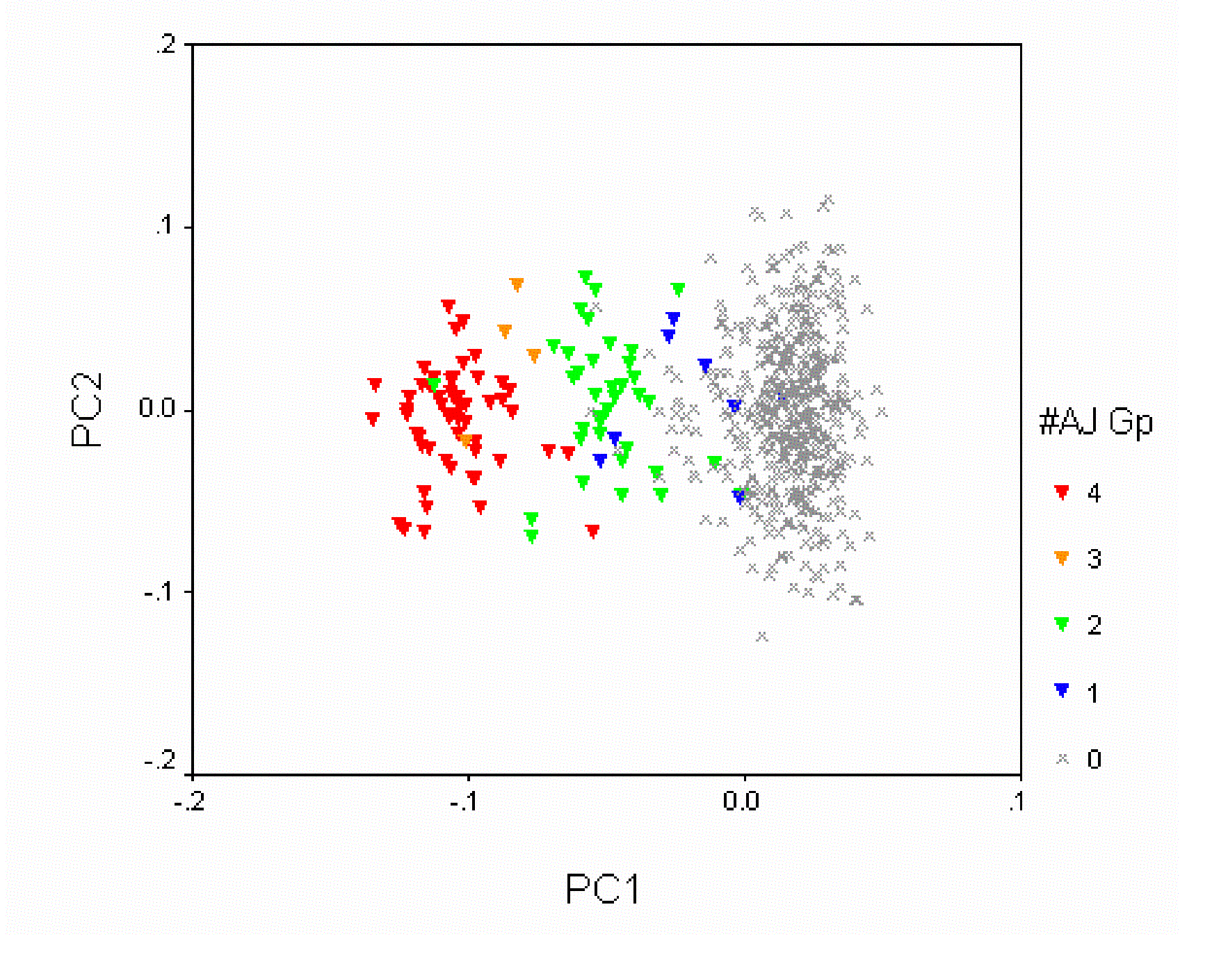

Supplement: Additional file 8 — Principal component analysis of Need et al. [21]cohort with designed Ashkenazi Jewish (AJ) specific ancestry informative markers. The numbers and the corresponding colors represent the degree of self-reported Ashkenazi admixture. [file gb-2012-13-1-r2-S8.BMP]

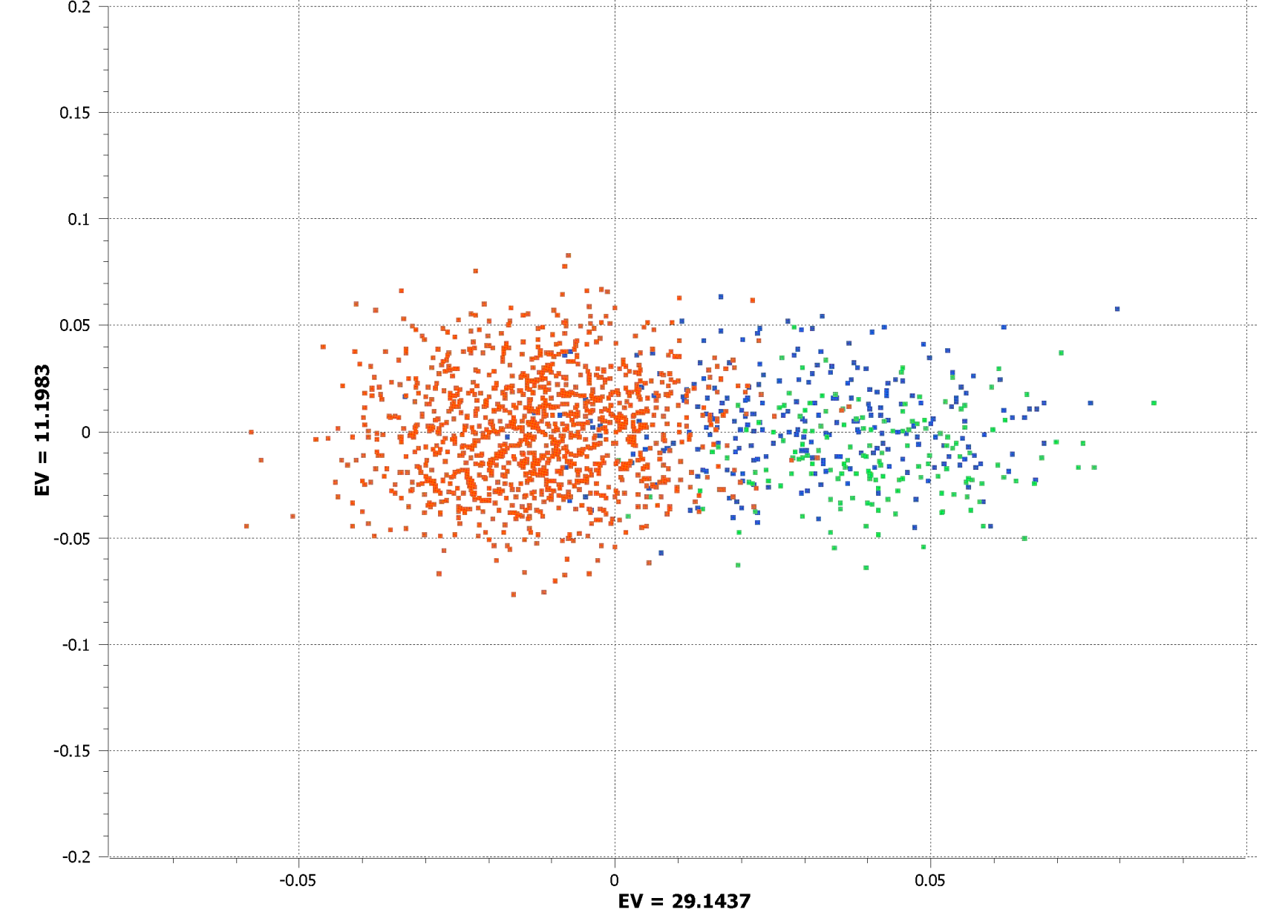

Supplement: Additional file 9 — Principal component analysis of all 1,312 AJ individuals with European HGDP individuals for 89 ancestry informative markers. Red indicates AJ individuals with C3 admixture score > 0.475, blue indicates AJ individuals with C3 admixture score < 0.475 and green indicates HGDP European individuals. [file gb-2012-13-1-r2-S9.BMP]
